# Effectiveness of Remote PPG Construction Methods: A Preliminary Analysis

**DOI:** 10.3390/bioengineering9100485

**Published:** 2022-09-20

**Authors:** Fridolin Haugg, Mohamed Elgendi, Carlo Menon

**Affiliations:** 1 Biomedical and Mobile Health Technology Lab, ETH Zurich, 8008 Zurich, Switzerland; 2 Department of Mechanical Engineering, Karlsruher Institute for Technology, 76131 Karlsruhe, Germany

**Keywords:** Imaging PPG, imaging photoplethysmogram, camera-based photoplethysmography, remote health monitoring, light interaction with tissue, vasomotor reactivity, vascular regulation, early diagnostic methods, imaging and sensing, optical imaging

## Abstract

The contactless recording of a photoplethysmography (PPG) signal with a Red-Green-Blue (RGB) camera is known as remote photoplethysmography (rPPG). Studies have reported on the positive impact of using this technique, particularly in heart rate estimation, which has led to increased research on this topic among scientists. Therefore, converting from RGB signals to constructing an rPPG signal is an important step. Eight rPPG methods (plant-orthogonal-to-skin (POS), local group invariance (LGI), the chrominance-based method (CHROM), orthogonal matrix image transformation (OMIT), GREEN, independent component analysis (ICA), principal component analysis (PCA), and blood volume pulse (PBV) methods) were assessed using dynamic time warping, power spectrum analysis, and Pearson’s correlation coefficient, with different activities (at rest, during exercising in the gym, during talking, and while head rotating) and four regions of interest (ROI): the forehead, the left cheek, the right cheek, and a combination of all three ROIs. The best performing rPPG methods in all categories were the POS, LGI, and OMI methods; each performed well in all activities. Recommendations for future work are provided.

## 1. Introduction

Photoplethysmography (PPG) is an optical measurement technique for estimating cardiovascular parameters such as heart rate and blood pressure [1,2]. PPG sensors are inexpensive and may easily be included in wearables; therefore, the number of studies investigating this issue have increased in recent years [3]. The underlying principle is simple: reflected light from certain regions of the skin is affected by the amount of blood under the skin. The captured light can then be used to measure blood volume changes. Remote PPG (rPPG) is the contactless measurement of the reflected light using a Red-Green-Blue (RGB) camera [4]. This low-cost method makes the recording of health-related data easier for many people to access because RGB cameras are often built into smartphones or laptops.

In the current body of literature, the rPPG signal is frequently compared solely to the extracted health-related information, such as heart rate or blood pressure, rather than to the ground truth PPG signal [5,6]. The error metrics used in this case are often the mean absolute error (MAE) or Pearson’s correlation coefficient (*r*) between the estimated and ground truth health-related information. This can be of limited help in determining whether the rPPG signal is of high quality because it only assesses the signal indirectly. The metrics most often used to compare the ground truth PPG with the estimated rPPG are the MAE or *r* of all sample points [7]. Furthermore, the PPG signal is occasionally evaluated with the rPPG signal via a frequency analysis or using the signal-to-noise ratio (SNR) [8]. However, with a reasonably long rPPG signal, sample noise and an offset are to be expected, leading to a high error and a low *r*, although to a human observer, the signal might seem very similar.

A lower-quality rPPG signal is often used for heart rate measurement since frequency analysis or peak detection algorithm are sufficient. A higher quality signal is required to determine more complex health-related information, such as diastolic or systolic blood pressure. The diastolic peak and notch are especially important for estimating health-related information that goes beyond heartbeat. To compare the quality of the signal of multiple rPPG methods, we used dynamic time warping (DTW), which, to the best of our knowledge, is new in this field.

The DTW algorithm is a popular alternative approach for comparing the similarities of different time series [9]. By allowing “elastic” transformation and time shifting, it has been proven to be extremely efficient in detecting similar shapes with different phases [10]. Furthermore, we performed a power spectrum (PS) analysis and compared the *r* of these two signals. In this study, we evaluated eight non-deep learning rPPG methods (plant-orthogonal-to-skin (POS), local group invariance (LGI), the chrominance-based method (CHROM), orthogonal matrix image transformation (OMIT), GREEN, independent component analysis (ICA), principal component analysis (PCA), and blood volume pulse (PBV) methods) and compared the similarities between the estimated rPPG and the reference fingertip RPPG signals using three evaluation metrics.

## 2. Methodology

**Figure 1:**
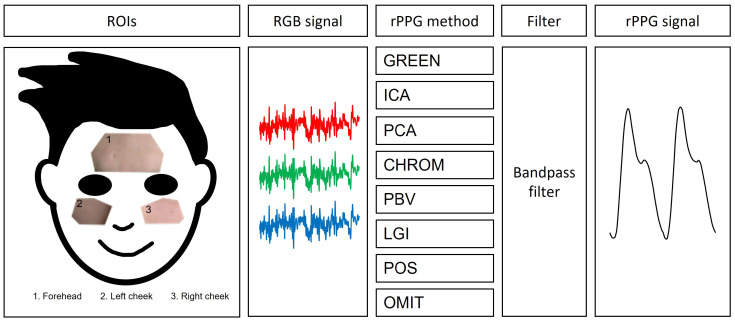
Graphical representation of the pipeline from a selfie video to an rPPG signal. Note that ROIs = region of interest, RGB signal = time series of average color channel value, rPPG method = method from the RGB signal to the rPPG signal.

For non-deep learning approaches, the procedure from the video to the rPPG signal has already been explained in detail by Boccignone et al. [11] In this paper, we will merely review the most significant parts of the procedure. The pipeline from the video to the rPPG signal is shown in Figure 1.

Due to blood volume changes, some areas of the human face influence the reflected light more than other areas. In this study, we evaluated some of the most frequently used ROIs for rPPG in the current literature: the right cheek, left cheek, and forehead [12,13]. Moreover, two independent ROI assessments from Sungjun et al. [14] and Dae-Yeol et al. [7] determined that the forehead and cheeks are the most promising ROIs for rPPG.

The landmarks that separate the ROIs from a frame that contains the face of the participant were detected with MediaPipe Face Mesh [15]. In the subsequent processing, the average value of the respective RGB channel was used. In this study, we evaluated four different ROIs. The forehead, left cheek, and right cheek were evaluated individually and then combined. The exact landmark numbers are as follows: forehead (107, 66, 69, 109, 10, 338, 299, 296, 336, 9), left cheek: (118, 119, 100, 126, 209, 49, 129, 203, 205, 50), and right cheek (347, 348, 329, 355, 429, 279, 358, 423, 425, 280).

The rPPG method is used to convert an RGB signal to an rPPG signal. All rPPG methods explored in the literature are listed in Table 1. It is important to note that principal component analysis (PCA) and independent component analysis (ICA) are rPPG methods based on blind source separation, in other words, without supervision or data labeling. In this study, the second component of ICA and PCA was used as the rPPG signal. All the mentioned rPPG methods were implemented in the Python framework for virtual heart rate and pyVHR, as reported in Boccignone et al. [16]. The present study used all the rPPG methods exactly as implemented in this framework. A wide variety of possible filters can be used to improve the rPPG signal. The present study aimed to assess different rPPG methods, not the optimal filter combination. Consequently, only a bandpass filter on the estimated rPPG signal was applied. The sixth-order bandpass filter ranged from 0.65 to 4 Hz.

### 2.1. Dataset

For the evaluation, the LGI-PGGI dataset from Pilz et al. [22] was applied. It contains video recordings with the participants’ faces in the center labelled with the referenced fingertip PPG signal. Videos from six participants, each with four different activities, are publicly available. The following activities are shown in the videos:

1 Resting. The participant is seated indoors with only minimal head movement.
2 Gym. The participant is doing an indoor workout on a bicycle ergometer.
3 Talk. The participant engaged in a conversation in an urban scenario with natural light.
4 Rotation. The participant made arbitrary head movements while indoors.

Each video is over 1 min in length. The pulse oximeter’s average sampling rate was 60 Hz, while the rate of the RGB camera was 25 Hz. A recent study [24] showed that a sampling rate of 25 Hz is sufficient for estimating heart rate.

### 2.2. Evaluation Metric

#### 2.2.1. DTW Distance

Under specific constraints, the goal of DTW is to provide a distance metric between two input time series by allowing “elastic” transformation and time shifting [10]. The distance metric is calculated by transforming the data into vectors and then computing the Euclidean distance between the points in vector space [10]. The present study used the software package DTAIDistance [25] for the DTW analysis.

**Figure 2:**
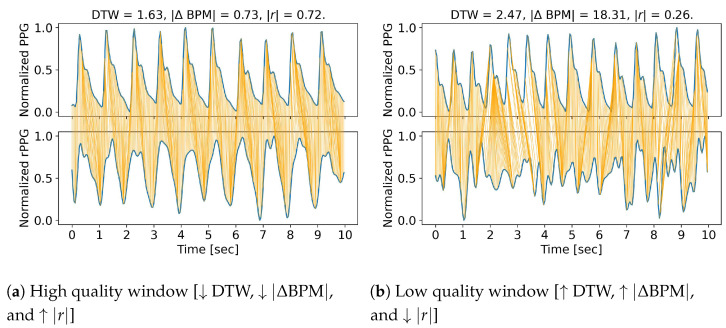
Comparison of a high quality window (**a**) and a low quality window (**b**). The high quality signal is from the participant named “Angelo” in the first window. It was recorded with the CHROM rPPG method at the Resting video activity with the forehead as the ROI: DTW = 1.63, |ΔBPM| = 0.73, and |r| = 0.72. The low quality signal is from the participant named “Harun” in the first window. It was recorded with the rPPG method ICA at the Resting video activity, with the left check as the ROI: DTW = 2.47, |ΔBPM| = 18.31, |r| = 0.26. Note that BPM = beats per minute, DTW = dynamic time warping, ICA = independent component analysis, rPPG = remote photoplethysmography, ROI = region of interest, |r| = correlation, OMIT = orthogonal matrix image transformation, PPG = photoplethysmography, ↑ = increase, and ↓ = decrease.

The average distance is calculated between a 10 s reference fingertip PPG signal and a 10 s rPPG window extracted from the video. The length of each video was cut to 1 min, resulting in six windows per video. Four different ROI cases, six participants with four different activities, and eight different rPPG methods, which created six windows per video, were evaluated, resulting in 4608 rPPG windows to compare with the reference fingertip PPG signal. As seen in Figure 2, the PPG signal and the reference fingertip rPPG signal window with a high similarity are compared to a PPG signal and the reference rPPG signal window with a low similarity.

#### 2.2.2. Beats-per-Minute Difference (ΔBPM)

**Figure 3:**
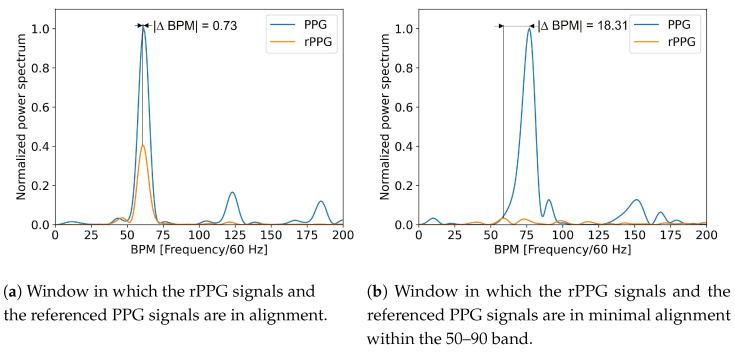
Comparison of a high quality window (**a**) and a low quality window (**b**) in the PS. The high quality signal is from the participant named “Angelo” in the first window. It was recorded with the CHROM rPPG method at the Resting video activity, with the forehead as the ROI: DTW = 1.63, |ΔBPM| = 0.73, and |r| = 0.72. The signal of low quality is from the participant named “Harun” in the first window. It was recorded with the ICA rPPG method at the Resting video activity, with the left cheek as the ROI: DTW = 2.47, |ΔBPM| = 18.31, |r| = 0.26. Note: BPM = beats per minute, DTW = dynamic time warping, ICA = independent component analysis, PS = power spectrum, rPPG = remote photoplethysmography, ROI = region of interest, |r| = correlation, OMIT = orthogonal matrix image transformation, and PPG = photoplethysmography.

The hlPS is commonly defined as the Fourier transformation of the autocorrelation function. This analysis is very popular for PPG and rPPG signals, as the peak in the PS graph corresponds to the heart rate. The frequency of the maximum in the PS graph from the PPG matches the heartbeat. In this study, we analyzed the absolute difference between the peak frequency of the rPPG signal and the PPG signal as an evaluation metric in the PS graph. As seen in Figure 3, the PS from the constructed rPPG and the reference fingertip PPG signal window with high similarity is compared to an rPPG signal and its referenced fingertip signal window with low similarity in the frequency domain.

#### 2.2.3. Correlation (*r*)

The *r* is calculated for each sampling point in a window

$$r=\frac{\sum_{i=1}^{n}x_{i}y_{i}-\sum_{i=1}^{n}x_{i}\sum_{i=1}^{n}y_{i}}{\sqrt{n\sum_{i=1}^{n}x_{i}^{2}-{\left(\sum_{i=1}^{n}y_{i}\right)}^{2}}\sqrt{n\sum_{i=1}^{n}y_{i}^{2}-{\left(\sum_{i=1}^{n}y_{i}\right)}^{2}}},$$

where $x_{i}$ are the sampling points of the PPG time series and $y_{i}$ are the sampling points of the rPPG time series. A 10 s window with a sampling rate of 25 Hz has 250 sampling points.

#### 2.2.4. Overall Evaluation Score

The overall evaluation score (OS) was calculated

$$\mathrm{OS}=\frac{1}{3}\left(\left(1-DTW_{n}\right)+\left(1-\Delta BPM_{n}\right)+r_{n}\right)$$

where $DTW_{n}$, $\Delta BPM_{n}$, and $r_{n}$ are the average values for all the ROI cases normalized between the eight rPPG methods. $DTW_{n}$, $\Delta BPM_{n}$, and $r_{n}$ are always in the range between 0 and 1.

## 3. Results

The PPG and rPPG signals were normalized with min–max normalization and compared with DTW, |ΔBPM|, and |r|. The results are shown below. After calculating the DTW, |ΔBPM|, and |r| per window of the signals, the mean of all six windows was calculated, followed by the mean for all six persons. All results in detail for each ROI case are shown in Table 2. The values have been rounded to two decimal digits for readability.

### 3.1. DTW Evaluation

#### 3.1.1. Different ROIs

**Figure 4:**
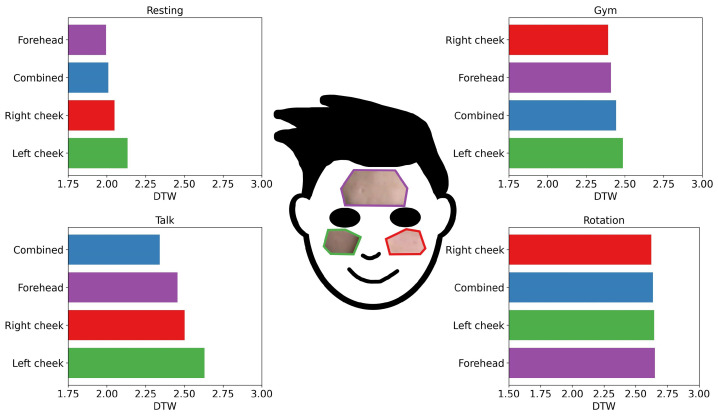
Comparison of DTW from the video activities Resting (top left), Gym (top right), Talking (bottom left), and Rotation (bottom right) for each ROI case. This chart shows the average of all the methods, participants, and windows for each investigated ROI. Note: DTW = dynamic time warping, ROI = region of interest.

The first objective was to evaluate the performance of the different rPPG methods for different ROIs. The more challenging video activities, Talking, Gym, and Rotation, were compared to the easiest activity, Resting (Figure 4).

With only minor movements in the Resting video activity, a smaller ROI, such as a forehead, can outperform the combined ROI approach. However, with more movement and natural lightning, as seen in the Talking video activity, the combined ROI case is best. In the natural light from the Talking video activity, the performance of the smaller ROIs, the left cheek and right cheek, was significantly worse than the performance of the forehead ROI. This result seems explicable because noise is less of a factor with a larger surface area.

Moreover, the landmark tracking of MediaPipe Face Mesh [15] was excellent for the forehead ROI, but there can be small shifts in the ROI on the cheeks with greater movement. For example, it is possible that with an extreme head position, the skin and background comprise the ROI of one of the cheeks (left or right cheek). In the Rotation video activity, it seems that every ROI was challenged in a similar way; there were no major differences in the ROIs for that activity.

#### 3.1.2. Different Video Activities

The second objective was to evaluate the performance of the different rPPG methods for different video activities. As seen in Table 2, Rotation was the most challenging video activity for DTW. That activity contains arbitrary and unnatural movements, followed by sections without movement, which makes it very difficult to create an rPPG signal. In this case, the errors of the applied landmark detection algorithm are an additional factor to consider due to fast and extreme movements.

**Figure 5:**
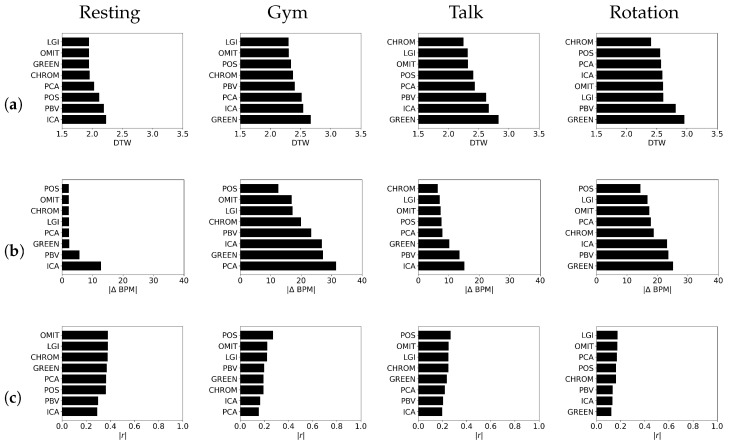
Average DTW (**a**), |ΔBPM| (**b**), |r| (**c**) over all participants, windows, and ROIs for every rPPG method. Note that BPM = beats per minutes, DTW = dynamic time warping, PPG = photoplethysmography, rPPG = remote photoplethysmography, |r| = correlation.

There is a unique ranking of the rPPG methods for each video activity. For the Resting and Gym video activities, LGI was the best method. For the Talking and Rotation video activities, the CHROM rPPG method showed particularly good results. For the difficult video activities, Gym and Talking, LGI was one of the two best performing methods. This data makes sense because earlier studies from the group behind developing LGI [22] have shown that the rPPG method has a high level of motion and lightning resilience in heart rate estimation. CHROM appeared to perform well in the natural light of the Talking video activity. In three out of four activities, GREEN was the worst method. Here, it can be observed that in different video activities, the various rPPG models performed differently, and there was no overall best model. However, it is easy to see that the worst results were obtained for activity with movement and natural lighting. The DTW results for different video activities are shown in Figure 5a.

#### 3.1.3. Beats-per-Minute Difference (ΔBPM)

As previously mentioned, a PS analysis was conducted. Only the first low-frequency envelope amplitude in the PS graph was compared because it determines the heart rate. The results are shown in Figure 5b. POS excelled in this area. It is particularly intriguing that the performance of POS significantly surpassed that of the other rPPG methods in the more difficult video activity, Gym. POS was found to be the best method in three of the four video activities. CHROM also showed a strong performance in the natural lightning in the Talking video activity. In the video activity Resting, it was found that the performance of the five best rPPG methods were similar; in fact, the differences in the performances were not significant. In two of the four video activities, ICA had the worst results.

#### 3.1.4. Correlation (*r*)

The achieved |r| between the rPPG signals and the reference fingertip PPG signals for all video activities can be seen in Figure 5c. A high correlation of (|r| > 0.7) for a single 10 s window was achieved. However, on average, it was very low, even for the best video activity, Resting. Notably, in this study, the window was not moved to avoid possible offsets because different frequencies made it impossible to significantly increase the |r| for the 1 minute recording. POS was one of the best methods in this comparison; it had the best performance in two of the four video activities: Gym and Talking. OMIT was also found to be one of the top two methods in every video activity. ICA was the worst-performing method in two out of four studied video activities.

### 3.2. Best rPPG Method Overall

**Figure 6:**
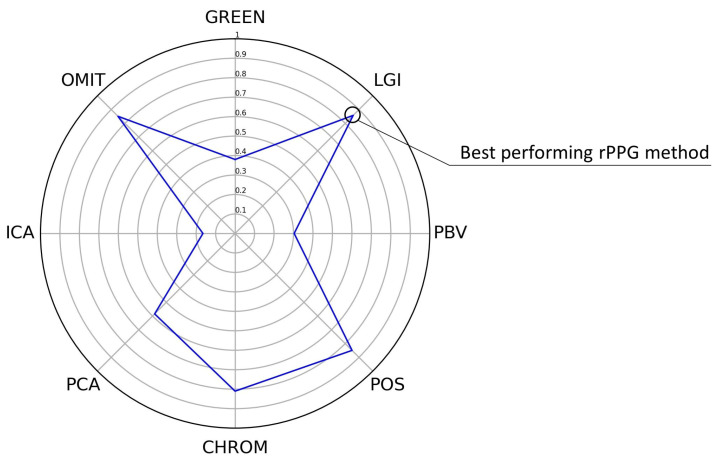
Average OS score for each rPPG method. Note that OS = overall evaluation score.

To find the best rPPG method overall, all values were normalized with min–max normalization, and the weighted sum was taken. DTW, |ΔBPM|, and |r| were weighted equally. The overall performance results are shown in Table 3 and Figure 6.

LGI, POS, and OMIT were the best overall methods for all the video activities combined, as seen in Figure 6. With a small advantage, LGI was the best method overall. For the Resting video activity with minimal movements, the rPPG methods LGI and OMIT showed the best results. In the Gym video activity, with a lot of movement and indoor lightning, the POS rPPG method performed particularly well. For natural lightning in the Talking video activity, CHROM was the best rPPG method. In the Rotation video activity, POS was again found to be the best rPPG method.

## 4. Discussion

This study demonstrated that none of the studied rPPG methods are the best for all the investigated cases. It has been shown that rPPG methods perform differently depending on the movement, the lighting conditions in the video, and the error metric that is applied. It is remarkable that in the Resting video activity, no major performance differences were found for the top five rPPG methods for BPM estimation; the differences in performance became greater in more challenging video activities. The performance of the POS rPPG method was the best overall among all the categories, and it was by far the best in the Gym video activity for BPM estimation. Thus, POS was the best rPPG method for BPM estimation in this study.

The performance of the POS rPPG method was superior to the other tested methods for the more difficult datasets with indoor lighting, such as the Gym and Rotation video activities. The great advantage of POS is that it is a mathematical model, which can be beneficial for medical applications to blind sourcing approaches. In the study by Boccoignone et al. [11], the POS rPPG method also showed superior performance for the LGI-PGGI dataset from Pilz et al. [22]. However, these results only apply to heartbeat estimation. The Talking video activity is of particular interest, as it was recorded under natural lighting. In our study, we also observed that the POS rPPG method had a good OS. Although very good results have already been achieved for heart rate estimation, it is clear from the |r| results that the rPPG is not yet a high quality signal; its quality is not equal to that of the reference fingertip PPG signal. When comparing the rPPG signal to the referenced PPG signal, it was discovered that the signals were still highly dissimilar, resulting in a low correlation. There are several factors that play a role here. When measuring a PPG signal with skin contact, there is significantly less noise. With rPPG, the signal is measured over a much larger area, which is why there is an average effect. The rPPG signal often does not have sharp systolic peaks; rather, the peaks are rounded. The quality of the rPPG signal heavily depends on the environment and movement conditions, which do not affect the PPG signal. Further research is needed to determine all the factors that influence an rPPG signal.

Windowing was performed on the RGB signal. Thus, blind source separation rPPG methods, such as PCA and ICA, could perform differently if windowing is applied to the rPPG signal. PCA and ICA try to find the most periodic signal in the RGB signal, which can lead to errors since motion can also be periodic, for example, in the Gym video activity. In this review, the ICA rPPG method did not show good results overall. The PBV method normalized the hole input color channels, which is why the windowing time also had a large influence. POS applies temporal normalization; therefore, in the POS method, a 10 s window starts and ends with a small amplitude. Eventually, the |r| and DTW could be higher if windowing is applied on the rPPG signal.

To determine the optimal ROI, further research is needed. Through the 458 landmarks in MediaPipe Face Mesh [15], the ROI can be determined accurately, and the tracking works very well. Many new ROIs can be easily tested. The size of the ROI is of particular interest; we assume that, under ideal conditions, a smaller ROI will result in a higher quality rPPG signal. We would like to point out that the LGI-PGGI dataset from Pilz et al. [22] has a bias and does not correspond to the general population. In that dataset, the prevailing ethnicity is Caucasian, which facilitates the creation of the PPG and rPPG signals [26]. No information was provided on the health status of the participants. Moreover, the public dataset only contains six people: five men and one woman. The participants are predominantly younger adults. Bias associated with race and gender is a well-known influencing factor in the literature [27,28]. However, related problems that intensify these issues also occur for rPPG methods [26]. The dataset employed in this study does not use specific lighting in front of the participants’ bodies, which is expected to increase the accuracy for every performance metric. Another important result of this study is that all the applied metrics have a comparable ranking. Well-performing methods frequently have a high ranking in all the applied metrics.

In the future, additional research is needed to obtain a high quality rPPG signal over a longer time window, which is suitable for blood pressure estimation or other health-related information. However, the technology of rPPG is very promising and can be beneficial, especially for a large population, because simple RGB cameras are installed in a variety of mobile devices. The recommendations derived from this study’s findings are summarized below:

1 We advise focusing research on optimal environmental conditions (minimal movement, constant light in front of the participant), as no high quality rPPG signal could be achieved with a good |r| over a longer time widow (>1 min).
2 We recommend using larger ROIs (such as forehead and cheeks) for challenging video activities (such as shifting background lights) and smaller ROIs (such as only a forehead) for easier activities.
3 We suggest using DTW as an error metric for comparing different ROIs, rPPG methods, and filters, because it handles time offsets very well, and it is very suitable for comparing signals from the different methods.
4 We advise using LGI, OMIT, or POS to obtain a high quality rPPG signal.

## 5. Conclusions

When comparing the rPPG signal to the referenced PPG signal, it was discovered that the constructed rPPG signals from RGB videos were highly dissimilar, resulting in a low correlation. However, comparing and ranking the rPPG construction methods is still possible. We demonstrated that different rPPG methods with different ROIs performed better or worse in different recording conditions. DTW was proven to be an effective technique for comparing various rPPG signals. In this study, the best-performing rPPG approaches were LGI, POS, and OMIT. In natural lighting conditions, larger ROIs showed better results than smaller ROIs. Future research is needed on the whole pipeline from facial video to rPPG; the impact of different filter combinations or ROI selection is still mainly unknown.

## Figures and Tables

**Table 1:** Summary of the rPPG methods.

rPPG Method	Summary
GREEN [17]	Of the three channels, the green channel is most like the PPG signal and can be used as its estimate.
ICA [18]	To recover three separate source signals, independent component analysis (ICA) is applied to the RGB signal. A significant rPPG signal was usually found in the second component.
PCA [19]	Principal component analysis (PCA) is applied to distinguish the rPPG signal from the RGB signal.
CHROM [20]	The chrominance (CHROM)-based method generates an rPPG signal by removing the noise caused by the light reflection using a ratio of the normalized color channels.
PBV [21]	PBV calculates the rPPG signal with blood volume pulse fluctuations in the RGB signal to identify the pulse-induced color changes from motion.
POS [8]	The plane-orthogonal-to-skin (POS) method uses the plane orthogonal to the skin tone in the RGB signal to extract the rPPG signal.
LGI [22]	The local group invariance (LGI) calculates an rPPG signal with a robust algorithm as a result of local transformations.
OMIT [23]	Orthogonal matrix image transformation (OMIT) recovers the rPPG signal by generating an orthogonal matrix with linearly uncorrelated components representing the orthonormal components in the RGB signal, relying on matrix decomposition.

**Table 2:** Evaluation metric results for constructing rPPG signals using eight methods. BPM = beats per minute, DTW = dynamic time warping, ROI = region of interest, *r* = correlation.

ROI	Metric	Video Activity	CHROM	LGI	POS	PBV	PCA	GREEN	OMIT	ICA
Forehead	DTW	Resting	1.85	1.91	2.07	2.11	1.98	1.92	1.91	2.20
		Gym	2.33	2.27	2.27	2.44	2.51	2.66	2.27	2.52
		Talk	2.11	2.27	2.40	2.59	2.45	2.79	2.27	2.78
		Rotation	2.44	2.56	2.59	2.84	2.63	2.96	2.51	2.68
	|r|	Resting	0.40	0.39	0.37	0.33	0.39	0.38	0.39	0.32
		Gym	0.21	0.24	0.29	0.21	0.16	0.19	0.24	0.16
		Talk	0.27	0.26	0.28	0.22	0.22	0.24	0.26	0.20
		Rotation	0.18	0.19	0.17	0.13	0.18	0.11	0.19	0.13
	|ΔBPM|	Resting	2.01	2.01	2.10	2.46	2.03	2.24	2.01	8.20
		Gym	16.38	11.03	7.57	23.38	29.01	25.55	11.01	29.01
		Talk	4.62	8.52	9.20	13.49	8.32	9.34	7.85	21.34
		Rotation	15.10	15.50	12.90	23.25	16.11	27.06	15.38	19.73
Left cheek	DTW	Resting	2.14	2.05	2.18	2.29	2.12	2.00	2.05	2.24
		Gym	2.51	2.36	2.42	2.36	2.56	2.69	2.37	2.62
		Talk	2.46	2.52	2.58	2.86	2.51	2.93	2.53	2.65
		Rotation	2.31	2.70	2.59	2.72	2.63	2.90	2.73	2.59
	|r|	Resting	0.35	0.37	0.36	0.24	0.35	0.34	0.37	0.25
		Gym	0.18	0.20	0.23	0.20	0.14	0.19	0.20	0.17
		Talk	0.21	0.21	0.24	0.18	0.20	0.21	0.21	0.17
		Rotation	0.15	0.17	0.15	0.15	0.17	0.13	0.17	0.14
	|ΔBPM|	Resting	2.22	2.22	2.18	13.39	2.28	2.99	2.22	11.70
		Gym	30.68	26.10	20.04	23.62	32.43	24.31	26.12	23.70
		Talk	12.57	7.71	6.10	16.28	8.44	11.41	7.67	16.01
		Rotation	21.97	18.66	14.91	23.80	19.31	23.15	19.25	26.61
Right cheek	DTW	Resting	1.96	1.95	2.14	2.12	2.06	1.93	1.95	2.28
		Gym	2.28	2.28	2.35	2.33	2.47	2.67	2.27	2.47
		Talk	2.36	2.36	2.41	2.54	2.44	2.86	2.36	2.68
		Rotation	2.46	2.60	2.49	2.79	2.45	2.94	2.62	2.62
	|r|	Resting	0.36	0.37	0.36	0.31	0.32	0.36	0.38	0.26
		Gym	0.17	0.21	0.26	0.17	0.15	0.19	0.21	0.16
		Talk	0.26	0.26	0.28	0.23	0.23	0.24	0.26	0.22
		Rotation	0.13	0.15	0.14	0.12	0.14	0.13	0.15	0.12
	|ΔBPM|	Resting	2.85	2.87	2.60	4.01	2.85	2.34	2.85	23.25
		Gym	18.01	19.29	15.46	23.44	35.87	31.51	19.31	28.65
		Talk	4.68	7.34	8.77	11.25	7.08	10.68	7.36	8.79
		Rotation	19.21	17.84	15.30	21.06	20.41	22.32	18.09	23.76
Combined	DTW	Resting	1.88	1.87	2.08	2.25	1.97	1.94	1.87	2.21
		Gym	2.38	2.30	2.32	2.47	2.53	2.67	2.30	2.57
		Talk	2.07	2.13	2.25	2.52	2.33	2.74	2.12	2.57
		Rotation	2.41	2.57	2.54	2.91	2.58	3.02	2.57	2.48
	|r|	Resting	0.41	0.39	0.36	0.32	0.40	0.41	0.39	0.34
		Gym	0.21	0.25	0.30	0.21	0.16	0.20	0.25	0.18
		Talk	0.27	0.27	0.27	0.19	0.23	0.25	0.27	0.20
		Rotation	0.18	0.19	0.19	0.14	0.19	0.13	0.19	0.14
	|ΔBPM|	Resting	1.91	1.91	1.99	2.95	1.93	2.03	1.87	8.10
		Gym	14.81	12.39	7.06	22.73	28.42	27.22	11.23	26.00
		Talk	3.68	4.52	6.37	13.08	7.91	9.09	6.39	14.16
		Rotation	18.84	15.12	14.44	26.16	15.60	28.06	16.72	22.38

**Table 3:** Average overall evaluation score for each rPPG method.

Video Activity	CHROM	LGI	POS	PBV	PCA	GREEN	OMIT	ICA
Resting	0.98	1.00	0.74	0.30	0.84	0.96	1.00	0.00
Gym	0.58	0.78	0.96	0.51	0.14	0.19	0.78	0.23
Talk	0.91	0.85	0.86	0.21	0.60	0.38	0.84	0.09
Rotation	0.78	0.80	0.83	0.20	0.76	0.00	0.78	0.34
Average	0.81	0.86	0.85	0.30	0.58	0.38	0.85	0.17

## Data Availability

The DTW algorithm can be downloaded from https://github.com/wannesm/dtaidistance/blob/master/docs/usage/dtw.rst (accessed on 15 August 2022). The dataset used is available at https://github.com/partofthestars/LGI-PPGI-DB. The applied rPPG methods are part of the pyVHR toolbox from https://github.com/phuselab/pyVHR.

## References

[1] Elgendi M. (2020). PPG Signal Analysis: An Introduction Using MATLAB^®^.

[2] Elgendi M., Fletcher R., Liang Y., Howard N., Lovell N.H., Abbott D., Lim K., Ward R. (2019). The use of photoplethysmography for assessing hypertension. NPJ Digit. Med..

[3] Bayoumy K., Gaber M., Elshafeey A., Mhaimeed O., Dineen E.H., Marvel F.A., Martin S.S., Muse E.D., Turakhia M.P., Tarakji K.G. (2021). Smart wearable devices in cardiovascular care: Where we are and how to move forward. Nat. Rev. Cardiol..

[4] Frey L., Menon C., Elgendi M. (2022). Blood pressure measurement using only a smartphone. NPJ Digit. Med..

[5] Steinman J., Barszczyk A., Sun H.S., Lee K., Feng Z.P. (2021). Smartphones and Video Cameras: Future Methods for Blood Pressure Measurement. Front. Digit. Health.

[6] Haugg F., Elgendi M., Menon C. (2022). Assessment of Blood Pressure Using Only a Smartphone and Machine Learning Techniques: A Systematic Review. Front. Cardiovasc. Med..

[7] Kim D.Y., Lee K., Sohn C.B. (2021). Assessment of ROI Selection for Facial Video-Based rPPG. Sensors.

[8] Wang W., den Brinker A.C., Stuijk S., de Haan G. (2016). Algorithmic Principles of Remote PPG. IEEE Trans. Biomed. Eng..

[9] Müller M. (2007). Dynamic time warping. Information Retrieval for Music and Motion.

[10] Senin P. (2008). Dynamic Time Warping Algorithm Review.

[11] Boccignone G., Conte D., Cuculo V., D’Amelio A., Grossi G., Lanzarotti R. (2020). An Open Framework for Remote-PPG Methods and Their Assessment. IEEE Access.

[12] Luo H., Yang D., Barszczyk A., Vempala N., Wei J., Wu S.J., Zheng P.P., Fu G., Lee K., Feng Z.P. (2019). Smartphone-Based Blood Pressure Measurement Using Transdermal Optical Imaging Technology. Circ. Cardiovasc. Imaging.

[13] Rong M., Li K. (2021). A Blood Pressure Prediction Method Based on Imaging Photoplethysmography in combination with Machine Learning. Biomed. Signal Process. Control.

[14] Kwon S., Kim J., Lee D., Park K. ROI analysis for remote photoplethysmography on facial video. Proceedings of the 2015 37th Annual International Conference of the IEEE Engineering in Medicine and Biology Society (EMBC).

[15] Kartynnik Y., Ablavatski A., Grishchenko I., Grundmann M. Real-time Facial Surface Geometry from Monocular Video on Mobile GPUs. Proceedings of the CVPR Workshop on Computer Vision for Augmented and Virtual Reality.

[16] Boccignone G., Conte D., Cuculo V., D’Amelio A., Grossi G., Lanzarotti R., Mortara E. (2022). pyVHR: A Python framework for remote photoplethysmography. PeerJ Comput. Sci..

[17] Verkruysse W., Svaasand L.O., Nelson J.S. (2008). Remote plethysmographic imaging using ambient light. Opt. Express.

[18] Poh M.Z., McDuff D.J., Picard R.W. (2010). Non-contact, automated cardiac pulse measurements using video imaging and blind source separation. Opt. Express.

[19] Lewandowska M., Rumiński J., Kocejko T., Nowak J. Measuring pulse rate with a webcam—A non-contact method for evaluating cardiac activity. Proceedings of the 2011 Federated Conference on Computer Science and Information Systems (FedCSIS).

[20] de Haan G., Jeanne V. (2013). Robust Pulse Rate From Chrominance-Based rPPG. IEEE Trans. Biomed. Eng..

[21] de Haan G., van Leest A. (2014). Improved motion robustness of remote-PPG by using the blood volume pulse signature. Physiol. Meas..

[22] Pilz C.S., Zaunseder S., Krajewski J., Blazek V. Local Group Invariance for Heart Rate Estimation from Face Videos in the Wild. Proceedings of the 2018 IEEE/CVF Conference on Computer Vision and Pattern Recognition Workshops (CVPRW).

[23] Casado C., López M. (2022). Face2PPG: An unsupervised pipeline for blood volume pulse extraction from faces. arXiv.

[24] Béres S., Hejjel L. (2021). The minimal sampling frequency of the photoplethysmogram for accurate pulse rate variability parameters in healthy volunteers. Biomed. Signal Process. Control.

[25] Meert W., Hendrickx K., Van Craenendonck T., Robberechts P. (2020). DTAIDistance. https://zenodo.org/record/3981067#.YywkCUxByUl.

[26] Dasari A., Prakash S.K.A., Jeni L.A., Tucker C.S. (2021). Evaluation of biases in remote photoplethysmography methods. NPJ Digit. Med..

[27] Sinaki F.Y., Ward R., Abbott D., Allen J., Fletcher R.R., Menon C., Elgendi M. (2022). Ethnic disparities in publicly-available pulse oximetry databases. Commun. Med..

[28] Elgendi M., Fletcher R., Tomar H., Allen J., Ward R., Menon C. (2021). The Striking Need for Age Diverse Pulse Oximeter Databases. Front. Med..

